# Serial bioelectrical impedance changes during nutritional follow-up in chronic pancreatitis

**DOI:** 10.3389/fgstr.2026.1933330

**Published:** 2026-10-20

**Authors:** Ilaria Lodola, Piera Zaccari, Martina Abati, Matteo Tacelli, Paolo Giorgio Arcidiacono, Gabriele Capurso

**Affiliations:** 1 Pancreatobiliary Endoscopy and Endosonography Division, IRCCS San Raffaele Scientific Institute, Milan, Italy; 2 Faculty of Medicine, Università Vita-Salute San Raffaele, Milan, Italy

**Keywords:** bioelectrical impedance analysis, body composition, chronic pancreatitis, nutritional counseling, phase angle

## Abstract

**Introduction:**

Nutritional impairment in chronic pancreatitis (CP) may be present even when body mass index (BMI) and routine screening scores do not indicate overt malnutrition. We aimed to provide a bioelectrical impedance analysis (BIA)-based snapshot of body composition in a clinically selected CP cohort referred for nutritional assessment and to evaluate serial changes during follow-up.

**Methods:**

This monocentric retrospective analysis included prospectively collected data from the BIOPANCREAS registry (NCT06552078). Adults with probable or definitive CP (M-ANNHEIM criteria) undergoing clinically indicated nutritional assessment including BIA were included. Longitudinal changes in BIA-derived parameters were assessed using linear mixed-effects models with Benjamini-Hochberg false discovery rate (FDR) correction. Complete-case Friedman analyses were performed as sensitivity analyses.

**Results:**

Thirty-two patients were included (mean age 58.4 ± 15.8 years; 65.6% male). At baseline, mean BMI was 22.3 ± 3.8 kg/m² and 77.4% were at low nutritional risk according to the Malnutrition Universal Screening Tool (MUST). Low fat-free mass index (FFMI) and appendicular skeletal muscle mass index (ASMMI) were identified in 18.8% and 40.6%, respectively. BIA measurements were available in 32, 25, and 17 patients at visits I, II, and III. After FDR correction, phase angle (PhA), body cell mass percentage (BCM%), and extracellular water percentage (ECW%) changed significantly. Compared with visit I, PhA increased by 0.24° at visit II (95% confidence interval [CI] 0.06–0.42) and 0.28° at visit III (95% CI 0.07–0.49); BCM% increased by 1.48 (95% CI 0.40–2.56) and 1.80 percentage points (95% CI 0.54–3.05), respectively; and ECW% decreased by 1.41 (95% CI −2.42 to −0.40) and 1.66 percentage points (95% CI −2.84 to −0.49), respectively; All six contrasts remained significant after FDR correction (adjusted p=0.016). No significant visit II to III differences or longitudinal changes in conventional muscle-quantity indices were observed.

**Discussion:**

In this real-world cohort, BIA identified heterogeneous body-composition abnormalities despite relatively reassuring BMI and MUST findings. During nutritional follow-up, PhA and BCM% increased and ECW% decreased, while conventional muscle-quantity indices did not show significant changes. These hypothesis-generating findings support prospective controlled evaluation of serial BIA during nutritional care in CP.

## Introduction

Chronic pancreatitis (CP) is a progressive fibro-inflammatory disease arising from genetic predisposition and/or environmental risk factors, characterized by an irreversible structural damage of the pancreas, resulting in progressive exocrine and endocrine dysfunction, recurrent abdominal pain, and impaired quality of life (1, 2).

Malnutrition and nutritional impairment are important systemic complications of CP and result from multiple, often coexisting mechanisms. Pancreatic exocrine insufficiency (PEI) may lead to maldigestion and nutrient malabsorption, while chronic abdominal pain, recurrent disease exacerbations, diabetes mellitus, alcohol misuse, and persistent systemic inflammation may contribute to reduced dietary intake and increased metabolic demands. Consequently, patients with CP are particularly vulnerable to progressive deterioration of their nutritional status, which has been associated with increased morbidity, poorer quality of life, and worse clinical outcomes. Current international guidelines therefore recommend that all patients with CP should undergo regular nutritional assessment to allow early identification of nutritional impairment (3, 4).

However, conventional anthropometric measures may not fully characterize nutritional status. Patients with CP may have a BMI within the normal range while presenting alterations in lean tissue, cellular characteristics and fluid distribution (5–7).

Assessment of body composition may therefore provide complementary information beyond body weight and BMI alone. Bioelectrical impedance analysis (BIA, 8, 9) represents a practical, less expensive, non-invasive bedside method that measures resistance and reactance and derives parameters such as phase angle (PhA), fat-free mass index (FFMI), appendicular skeletal muscle mass index (ASMMI), body cell mass (BCM), and extracellular water (ECW).

These variables capture different domains of nutritional status, including muscle quantity, metabolically active tissue, cellular integrity, and fluid distribution, and several have shown prognostic associations in chronic disease (10, 11, 12, 13, 14, 15, 16).

Some BIA-derived variables may change over relatively short periods of nutritional care. Cornejo-Pareja et al. reported a real-world cohort of 283 malnourished or nutritionally at-risk adults receiving dietary counselling and oral nutritional supplementation for up to six months: body weight and fat-free mass increased overall, while in the subgroup with serial BIA the HMB-enriched high-protein supplement was associated with increases in PhA and BCM (17). Although that population and intervention differ from CP, these findings support the hypothesis that qualitative BIA markers may be responsive to nutritional management before clear changes in conventional muscle-mass indices become evident.

Interpretation of BIA also requires caution because reference values and thresholds depend on measurement method, age, sex, device, and clinical population; for PhA in particular, contemporary reference data are age- and sex-specific rather than based on a single universal cut-off (18). We therefore aimed to provide a real-world snapshot of BIA-derived body-composition characteristics in a clinically selected CP cohort referred for nutritional assessment, to describe the frequency of reduced FFMI and ASMMI using published sex-specific thresholds, and to evaluate serial changes in BIA-derived parameters during routine nutritional follow-up.

## Materials and methods

### Study design and population

This retrospective analysis of prospectively collected data was conducted at IRCCS San Raffaele Hospital (Milan, Italy), a tertiary referral center for pancreatic diseases, between March 2022 and May 2024. Patients with CP were prospectively enrolled in the BIOPANCREAS registry (ClinicalTrials.gov: NCT06552078).

The present analysis included adult patients (age≥18 years) with probable or definitive CP according to the M-ANNHEIM classification (19) who underwent a dedicated nutritional assessment including bioelectrical impedance analysis (BIA). Nutritional assessment with BIA was not protocol-mandated for all patients enrolled in BIOPANCREAS but was performed according to clinical practice when concern regarding nutritional status prompted dedicated nutritional evaluation and follow-up. Previous pancreatic surgery was not an exclusion criterion.

Clinical and demographic data were prospectively collected during outpatient visits, hospital admissions or scheduled endoscopic procedures. CP etiology was classified according to TIGAR-O classification (20). Baseline variables included age, sex, smoking history, alcohol exposure, disease duration, previous pancreatic surgery, pancreatic calcifications, exocrine pancreatic insufficiency (PEI), endocrine pancreatic insufficiency and imaging findings. EUS findings compatible with chronic pancreatitis were defined according to the Rosemont classification (21). Patients with a previous history of alcohol exposure had discontinued active alcohol abuse before the first nutritional assessment.

PEI was assessed with faecal elastase-1 (mcg/gr) defining PEI under 200 mcg/gr and severe PEI under 100 mcg/gr (22, 23). Pancreatic endocrine insufficiency was defined when patients had an HbA1c value ≥6.5% or a fasting blood glucose ≥ 126 mg/dl, or a 2 h post-oral glucose tolerance test glucose level ≥ 200 mg/dl (24).

All patients with pancreatic exocrine insufficiency were receiving pancreatic enzyme replacement therapy (PERT), with treatment dose reviewed and optimized as clinically indicated at each nutritional assessment. Patients also received standard-of-care management for chronic pancreatitis and its complications, including treatment of pain and diabetes when present, together with counselling regarding alcohol abstinence and smoking cessation.

### Nutritional assessment in CP

Nutritional assessment was performed during dedicated outpatient nutritional consultations by a biologist nutritionist and included nutritional screening, anthropometric measurements, dietary assessment, body composition analysis and pain assessment. Nutritional management was conducted according to routine clinical practice and current ESPEN recommendations for chronic pancreatitis (3, 25).

Nutritional risk at study entry was assessed using the Malnutrition Universal Screening Tool (MUST), when available, as part of the routine medical assessment preceding referral for dedicated nutritional evaluation. MUST classifies patients as being at low (score 0), medium (score 1), or high nutritional risk (score ≥2) based on BMI, unintentional weight loss, and the effect of acute disease on nutritional intake. MUST data were used descriptively and were not intended to establish a formal diagnosis of malnutrition (26).

Body weight (kg) and height (cm) were measured at baseline, and BMI (kg/m²) was calculated as weight/height^2^ (Kg/m^2^). Mid-upper arm circumference (MUAC) and calf circumference (CC) were measured with a non-elastic tape, with MUAC measured at the midpoint between the acromion and olecranon, and CC at the point of maximum circumference of the non-dominant calf. These circumference measures were used descriptively and as supportive indicators of muscle quantity (27).

Dietary intake was assessed through a structured dietary interview. Pain severity was evaluated using a 100-mm visual analogue scale (VAS) ranging from 0 (no pain) to 100 (worst imaginable pain).

#### Body composition analysis

Body composition was assessed by single-frequency bioelectrical impedance analysis (BIA; Akern, Florence; Italy, 50 kHz). All examinations were performed by the same biologist nutritionist. Patients were assessed fasting, were asked to empty their bladder approximately 30 minutes before the measurement and remained in the supine position for at least 15 minutes before acquisition. A standard tetrapolar electrode configuration was applied to the right side of the body, with electrodes positioned on the hand and foot according to standard recommendations. No patient had an implanted cardiac pacemaker.

Resistance (R) and reactance (Xc) were obtained as raw impedance measurements, and phase angle (PhA) was derived from R and Xc. The remaining body-composition parameters including body cell mass (BCM), fat-free mass (FFM), appendicular skeletal muscle mass (ASMM), skeletal muscle mass (SMM), fat mass (FM), total body water (TBW), and extracellular water (ECW) were estimated from impedance measurements using validated predictive equations incorporated into the device software.

BCM% was expressed as BCM relative to FFM (BCM/FFM ×100), whereas ECW% was expressed as ECW relative to TBW (ECW/TBW ×100).

Published sex-specific thresholds were used to describe reduced muscle quantity according to FFMI (<17 kg/m² in men and <15 kg/m² in women) and ASMMI (<7.0 kg/m² in men and <5.5 kg/m² in women, 28). These thresholds were used descriptively and were not considered diagnostic of sarcopenia, which requires assessment of muscle strength and/or physical performance. Because validated CP-specific cut-offs are not available and PhA is strongly dependent on age, sex, and measurement characteristics, PhA and BCMI were analyzed as continuous variables (18).

#### Nutritional management and follow-up

Nutritional management was individualized according to routine clinical practice and current ESPEN recommendations for chronic pancreatitis. Estimated energy requirements were generally set at approximately 30–35 kcal/kg ideal body weight/day and protein requirements at 1.2–1.5 g/kg ideal body weight/day, with individual adjustment according to nutritional status and clinical needs.

In patients with insufficient energy or protein intake, nutritional counselling aimed to increase dietary energy and protein intake, and oral nutritional supplements (ONS) were prescribed when dietary measures alone were considered insufficient to meet estimated requirements. ONS formulation and dose were individualized to bridge the estimated energy and protein gap in accordance with ESPEN recommendations and were reassessed at follow-up; product choice and duration were therefore not protocolized as a uniform intervention. In patients without evidence of nutritional depletion, counselling focused on maintaining a balanced diet with adequate protein intake. Dietary fat was not routinely restricted, except when clinically indicated according to symptoms and tolerance.

Dietary recommendations were provided at the first nutritional consultation and reassessed during follow-up through dietary recall and clinical interview. No standardized dietary adherence questionnaire was used. Follow-up nutritional assessments were performed according to routine clinical practice rather than at protocol-mandated intervals and repeat BIA measurements were obtained when patients returned for reassessment. The nutritional visits generally occurred between scheduled gastroenterology follow-up assessments, which in routine practice were approximately annual; no changes in analgesic treatment by the gastroenterology team were recorded during the nutritional follow-up interval. Reasons for missed nutritional follow-up visits were not systematically recorded.

### Statistical analysis

Continuous variables were tested for normality using the Shapiro-Wilk test and homogeneity of variances was assessed using Levene’s test. Normally distributed data were presented as mean ± standard deviation (SD), while categorical variables are reported as frequencies and percentages.

Comparisons between groups were performed using Student’s t-test (with Welch’s correction when the assumption of equal variances was not met) for continuous variables and Fisher’s exact test for categorical variables. Bivariate correlations between continuous variables were evaluated using Pearson’s correlation coefficient and visualized using a correlation plot. Baseline cut-off-based subgroup comparisons were considered exploratory; where multiple related comparisons were performed, Benjamini-Hochberg false discovery rate (FDR) correction was applied.

Longitudinal changes in BIA-derived parameters were assessed using separate linear mixed-effects models, with visit included as a categorical fixed effect and patient as a random intercept. All available measurements were included in the models.

The overall effect of visit was tested separately for each of the 15 longitudinal BIA-derived parameters. To account for multiple testing, p values from the 15 overall visit tests were adjusted using the Benjamini-Hochberg false discovery rate (FDR) procedure. For parameters showing a significant overall effect after FDR correction, model-based pairwise comparisons between visits were performed and estimated mean differences were reported with 95% confidence intervals. The Benjamini-Hochberg procedure was also applied across these pairwise comparisons.

To explore whether longitudinal changes in phase angle could primarily reflect changes in hydration status, an exploratory analysis of the raw impedance components was performed (29). Resistance and reactance were normalized by height (R/H and Xc/H, Ω/m), and impedance vector length was calculated as Z/H. Longitudinal changes in these variables were assessed using the same linear mixed-effects model structure applied to the other BIA-derived parameters, with visit as a categorical fixed effect and patient as a random intercept. Benjamini-Hochberg FDR correction was applied across the three global tests. For any raw impedance component showing a significant overall effect, model-based pairwise contrasts were performed, with Benjamini-Hochberg FDR correction across the three pairwise comparisons.

As a sensitivity analysis, complete-case Friedman tests were performed for all 15 BIA-derived parameters in patients with measurements available at all three visits. FDR correction was applied across the 15 sensitivity analyses.

For graphical presentation, individual longitudinal patient trajectories (spaghetti plot) with jittered observations and model-estimated marginal means with 95% confidence intervals were displayed for phase angle, BCM%, and ECW%.

To explore concurrent clinical changes that might contribute to the observed BIA pattern, longitudinal body weight, BMI, pain VAS, and total daily PERT exposure were analyzed using separate linear mixed-effects models fitted using restricted maximum likelihood (REML), with visit as a categorical fixed effect and patient as a random intercept. Analyses of pain and PERT were restricted to visits with concomitant BIA. For PERT exposure, patients documented as not receiving PERT were assigned 0 U/day, whereas observations with missing PERT status or missing dose among PERT users were treated as missing. Benjamini-Hochberg FDR correction was applied across the four overall visit tests. For outcomes showing a significant overall effect after FDR correction, model-based pairwise contrasts were performed and FDR correction was applied across the resulting pairwise comparisons.

All statistical analyses were performed using R software. A two-sided p-value<0.05 was considered statistically significant for hypothesis testing.

## Results

### Baseline characteristics of the overall cohort

During the study period, 282 CP patients were enrolled in the BIOPANCREAS registry. Of these, 40 patients (14.2%) were referred for dedicated nutritional assessment including BIA according to clinical indication, whereas 242 patients were not referred. Among the 40 referred patients, 6 did not attend the scheduled nutritional assessment and 2 underwent nutritional assessment without BIA. Therefore, 32 patients (11.3% of the overall registry) underwent dedicated nutritional evaluation including baseline BIA according to clinical indication and were included in the present analysis (**Figure 1**).

**Figure 1:**
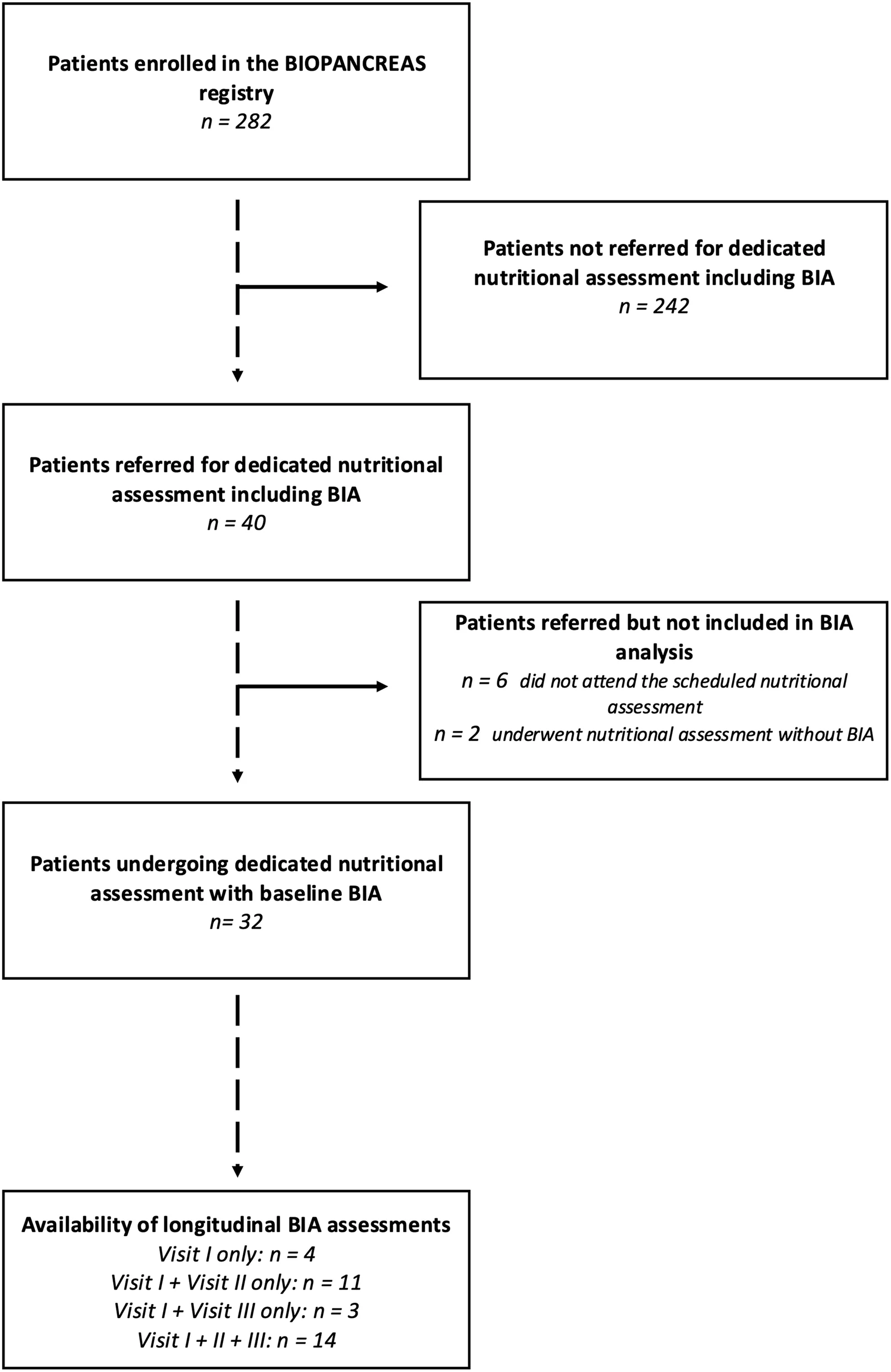
Study flow diagram: flow of patients from the overall BIOPANCREAS registry to the cohort included in the present bioelectrical impedance analysis (BIA), with availability of longitudinal nutritional and BIA assessments across follow-up visits.

The study cohort was predominantly male (65.6%) with a mean age of 58.4 ± 15.8 years. The most common etiology was exotoxic/alcohol-related chronic pancreatitis (53.1%), followed by genetic (15.6%), autoimmune (12.5%), and idiopathic disease (9.4%). Smoking exposure was reported by 81.3% of patients, while 58.1% had a history of alcohol exposure. No patient had ongoing alcohol abuse at the time of the first nutritional assessment.

The population was characterized by advanced chronic pancreatitis, as reflected by the high prevalence of exocrine pancreatic insufficiency (90.6%), severe exocrine pancreatic insufficiency (56.3%), pancreatic calcifications (78.1%), and EUS features compatible with chronic pancreatitis according to the Rosemont criteria (84.4%). Previous pancreatic surgery had been performed in 7 patients (21.9%), whereas endocrine pancreatic insufficiency was present in 40.6% (**Table 1**).

**Table 1:** Baseline demographic and clinical characteristics of the study population (N = 32).

Baseline characteristic	Mean ± SD/n(%)
Mean Age, years	58.4 ± 15.8
Male sex, n (%)	21 (65.6)
Duration of disease before assessment, years	6.7 ± 6.7
Ever Smoking, n (%)	26 (81.3)
Alcohol exposure, n (%)	18/31 (58.1)
BMI at diagnosis, kg/m²	24.0 ± 3.7
BMI at first nutritional assessment, kg/m²	22.3 ± 3.8
Previous pancreatic surgery, n (%)	7 (21.9)
Exocrine pancreatic insufficiency (PERT-treated), n (%)	29 (90.6)
Severe exocrine pancreatic insufficiency, n (%)	18 (56.3)
Endocrine pancreatic insufficiency, n (%)	13 (40.6)
Pancreatic calcifications, n (%)	25 (78.1)
Osteopenia/osteoporosis, n (%)	9 (28.1)
MUST score ≥1, n (%)	7/31 (22.6)

Values are reported as mean ± standard deviation or n (%).

BMI, Body Mass Index; MUST Malnutrition Universal Screening Tool.

At the first nutritional consultation, mean BMI was 22.3 ± 3.8 kg/m², representing a decline from both usual BMI before diagnosis (23.9 ± 3.0 kg/m²) and BMI at diagnosis (24.0 ± 3.7 kg/m²). Baseline MUST was available in 31 patients: 24 (77.4%) were classified as low nutritional risk, 4 (12.9%) as medium risk and 3 (9.7%) as high risk.

### Baseline bioelectrical impedance parameters and frequency of abnormalities

Baseline bioelectrical impedance analysis demonstrated a mean phase angle of 5.0 ± 0.7°, BCMI of 8.7 ± 1.7 kg/m², FFMI of 18.0 ± 2.6 kg/m², and ASMMI of 6.6 ± 1.1 kg/m².

Mean ECW/TBW (ECW%) was 51.1 ± 3.8%, while mean BCM/FFM (BCM%) was 48.0 ± 4.0%. Additional BIA-derived parameters are summarized in **Table 2**.

**Table 2:** Nutritional and body composition parameters at the first nutritional consultation.

Parameter	Mean ± SD
Weight loss during previous 3–6 months (%)	7.4 ± 8.6
Pain VAS score	68.4 ± 28.6
Bioimpedance and Nutritional variables	
Resistance (Ω)	537.3 ± 104.6
Reactance (Ω)	46.7 ± 9.8
Phase angle (°)	5.0 ± 0.7
TBW (L)	37.6 ± 7.2
TBW % (%)	60.5 ± 6.8
ECW (L)	19.3 ± 3.7
ECW % (%)	51.1 ± 3.8
BCM (kg)	24.4 ± 5.4
BCM % (%)	48.0 ± 4.0
BCMI (kg/m²)	8.7 ± 1.7
FM (kg)	12.4 ± 6.4
FM % (%)	19.2 ± 8.1
FMI (kg/m²)	4.5 ± 2.4
FFM (kg)	50.7 ± 9.3
FFM % (%)	81.5 ± 8.8
FFMI (kg/m²)	18.0 ± 2.6
Skeletal muscle mass (kg)	25.2 ± 5.9
SMI (kg/m²)	9.0 ± 1.6
ASMM (kg)	18.8 ± 4.0
ASMMI (kg/m²)	6.6 ± 1.1
Waist circumference (cm)	84.7 ± 11.4
Hip circumference (cm)	93.1 ± 7.4
Mid-upper arm circumference (cm)	27.1 ± 4.2
Calf circumference (cm)	34.3 ± 2.8
Waist-to-hip ratio	0.91 ± 0.09

Values are reported as mean ± standard deviation.

VAS, Visual Analogue Scale; TBW, Total Body Water; ECW, Extracellular Water; BCM, Body Cell Mass; BCMI, Body Cell Mass Index; FM, Fat Mass; FMI, Fat Mass Index; FFM, Fat-Free Mass; FFMI, Fat-Free Mass Index; SMI, Skeletal Muscle Index; ASMM, Appendicular Skeletal Muscle Mass; ASMMI, Appendicular Skeletal Muscle Mass Index.

Using the predefined sex-specific thresholds for reduced muscle quantity, low FFMI was present in 6 of 32 patients (18.8%) and low ASMMI in 13 of 32 patients (40.6%). PhA and BCMI were not dichotomized because validated CP-specific cut-offs were not available. Accordingly, BIA did not simply identify uniformly abnormal body composition, but revealed a more heterogeneous profile, with reduced appendicular muscle quantity being relatively frequent despite a normal mean BMI and predominantly low MUST risk.

Exploratory comparisons did not identify a consistent pattern of association between BIA-derived abnormalities and age, pain severity, smoking exposure, or CP etiology after correction for multiple testing. Current BMI was lower among patients with low FFMI and ASMMI (FDR-adjusted q<0.05); given the intrinsic relationship between body size and these body-composition indices, these comparisons were considered descriptive.

Correlation analysis of baseline bioelectrical impedance parameters revealed strong positive associations among markers of metabolically active and muscle mass, including BCMI, FFMI, SMI, ASMMI, and phase angle. Conversely, extracellular water-related parameters clustered separately and showed inverse relationships with indices of cellular integrity and nutritional status. These findings suggest that bioelectrical impedance analysis captures distinct but biologically coherent domains reflecting cellular health, body composition, and hydration status. A complete correlation matrix is provided in **Figure 2**.

**Figure 2:**
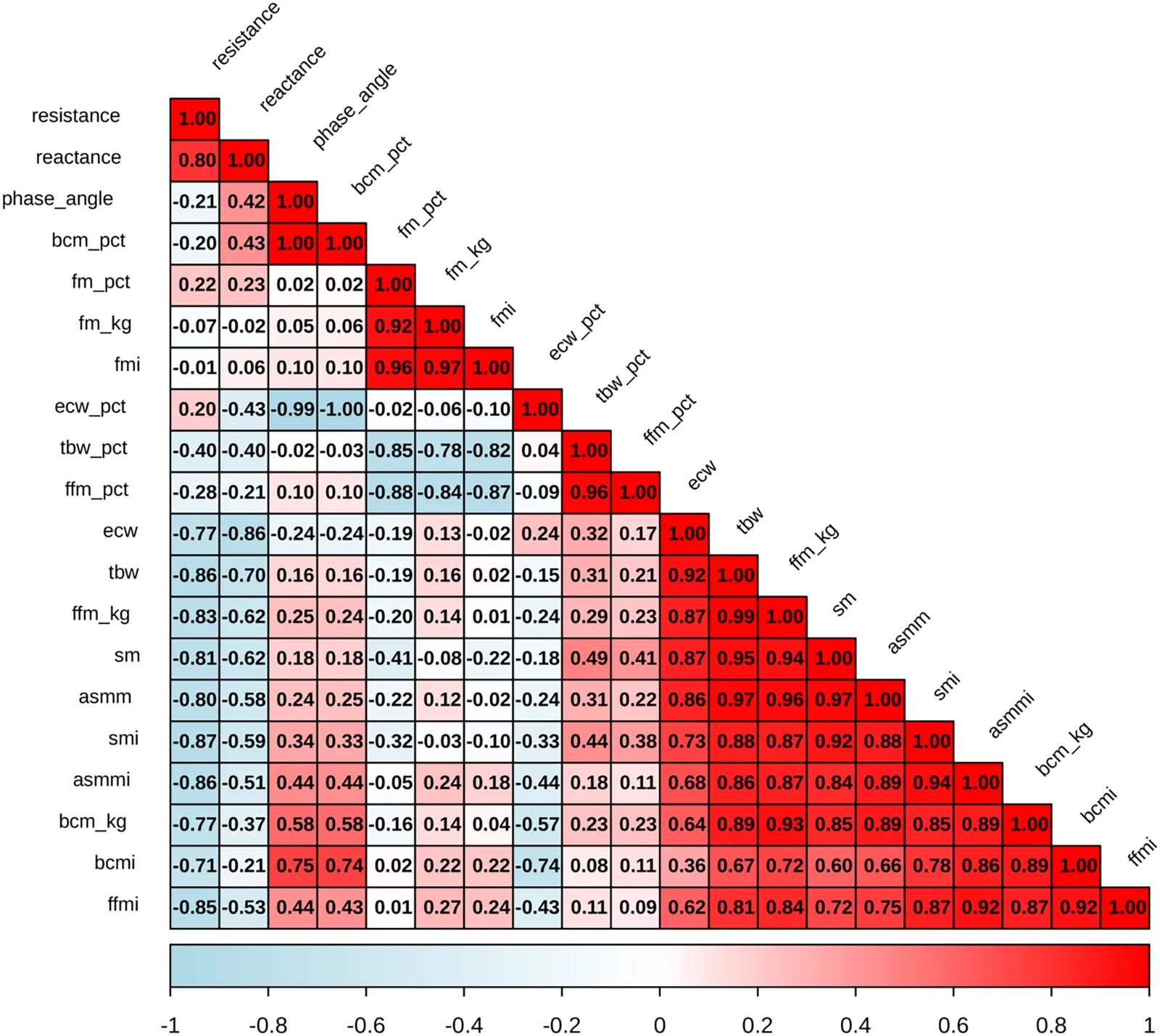
Pearson correlation matrix of baseline bioelectrical impedance-derived parameters in the overall chronic pancreatitis (CP) cohort. Positive correlations are shown in red and negative correlations in blue. The intensity of the colour reflects the strength of the association, with more intense hues indicating stronger correlations.

### Longitudinal changes in bioelectrical impedance parameters

Among the 32 patients included, BIA measurements were available for 25 patients (78.1%) at visit II and 17 patients (53.1%) at visit III. Fourteen patients (43.8%) had completed BIA measurements at all three visits, whereas three patients underwent visits I and III without an available visit II assessment (**Figure 1**). Nutritional management consisted of dietary counseling alone in 14 patients (43.7%) and dietary counseling combined with oral nutritional supplementation (ONS) in 18 patients (56.3%). Among patients completing all three assessments, 10 (71.4%) received ONS, whereas 4 (28.6%) received dietary counseling alone.

The median interval between the first and second nutritional visits was 2.99 months (IQR 2.30–3.22), while the median interval between the second and third visits was 2.99 months (IQR 2.40–3.45).

In the linear mixed-effects analyses, significant longitudinal changes after FDR correction were observed for ECW% (FDR-adjusted p=0.030), BCM% (FDR-adjusted p=0.030), and PhA (FDR-adjusted p=0.033, **Table 3**). No statistically significant longitudinal changes were detected in conventional indices of muscle quantity, including FFMI, SM, SMI, ASMM, and ASMMI. Raw reactance also showed an overall visit effect after FDR correction and is reported in **Table 3** but was not interpreted separately because PhA is derived from resistance and reactance.

**Table 3:** Longitudinal mixed-effects analysis of BIA-derived parameters.

Outcome	Visit I estimated mean (95% CI)	Visit II estimated mean (95% CI)	Visit III estimated mean (95% CI)	Δ Visit II − I (95% CI)	Δ Visit III − I (95% CI)	Overall p	FDR-adjusted p
*Resistance (Ω)*	537.3 (504.6 to 570.1)	539.7 (506.1 to 573.4)	523.6 (488.2 to 558.9)	+2.40 (-15.50 to +20.30)	-13.76 (-34.64 to +7.12)	0.317	0.419
*Reactance (Ω)*	46.68 (43.35 to 50.01)	49.34 (45.91 to 52.77)	48.75 (45.16 to 52.35)	+2.66 (+0.85 to +4.47)	+2.07 (-0.04 to +4.18)	**0.010**	**0.038**
*Phase angle (°)*	5.00 (4.78 to 5.22)	5.24 (5.01 to 5.48)	5.28 (5.02 to 5.54)	+0.244 (+0.063 to +0.425)	+0.280 (+0.069 to +0.491)	**0.007**	**0.033**
*TBW (%)*	60.28 (58.06 to 62.50)	58.84 (56.54 to 61.14)	59.23 (56.79 to 61.67)	-1.44 (-2.81 to -0.07)	-1.05 (-2.65 to +0.54)	0.101	0.217
*ECW (%)*	51.11 (49.87 to 52.34)	49.69 (48.38 to 51.00)	49.44 (48.00 to 50.88)	-1.414 (-2.424 to -0.405)	-1.663 (-2.840 to -0.486)	**0.004**	**0.030**
*BCM (%)*	47.99 (46.68 to 49.30)	49.47 (48.08 to 50.86)	49.78 (48.25 to 51.31)	+1.481 (+0.405 to +2.557)	+1.796 (+0.541 to +3.050)	**0.004**	**0.030**
*BCMI (kg/m²)*	8.66 (8.12 to 9.21)	8.94 (8.37 to 9.50)	9.09 (8.49 to 9.69)	+0.27 (-0.06 to +0.61)	+0.43 (+0.04 to +0.81)	0.069	0.207
*FM (%)*	19.38 (16.65 to 22.12)	19.93 (17.10 to 22.76)	19.74 (16.74 to 22.74)	+0.55 (-1.09 to +2.19)	+0.36 (-1.56 to +2.27)	0.800	0.800
*FMI (kg/m²)*	4.49 (3.69 to 5.28)	4.69 (3.88 to 5.51)	4.64 (3.79 to 5.49)	+0.20 (-0.20 to +0.61)	+0.15 (-0.32 to +0.62)	0.584	0.625
*FFM (%)*	81.29 (78.46 to 84.12)	79.89 (76.97 to 82.81)	79.89 (76.81 to 82.97)	-1.40 (-3.04 to +0.23)	-1.41 (-3.31 to +0.50)	0.163	0.271
*FFMI (kg/m²)*	17.97 (17.13 to 18.80)	17.98 (17.13 to 18.83)	18.22 (17.33 to 19.10)	+0.02 (-0.36 to +0.40)	+0.25 (-0.19 to +0.70)	0.505	0.583
*SM (kg)*	25.19 (23.25 to 27.12)	24.81 (22.85 to 26.78)	25.50 (23.49 to 27.51)	-0.37 (-1.08 to +0.33)	+0.31 (-0.51 to +1.13)	0.282	0.419
*SMI (kg/m²)*	8.90 (8.36 to 9.43)	8.79 (8.24 to 9.33)	9.01 (8.45 to 9.58)	-0.11 (-0.36 to +0.14)	+0.12 (-0.17 to +0.41)	0.335	0.419
*ASMM (kg)*	18.77 (17.46 to 20.07)	18.83 (17.51 to 20.15)	19.27 (17.92 to 20.61)	+0.06 (-0.36 to +0.49)	+0.50 (+0.00 to +1.00)	0.129	0.242
*ASMMI (kg/m²)*	6.64 (6.29 to 6.99)	6.67 (6.31 to 7.02)	6.83 (6.46 to 7.19)	+0.03 (-0.12 to +0.18)	+0.19 (+0.01 to +0.36)	0.098	0.217

Values at each visit are model-estimated marginal means with 95% confidence intervals from separate linear mixed-effects models fitted using restricted maximum likelihood (REML), with visit as a categorical fixed effect and patient as a random intercept. Δ values represent model-estimated contrasts versus visit I. The overall p value tests the global effect of visit. Benjamini-Hochberg FDR correction was applied across the 15 overall visit tests.

TBW, Total Body Water; ECW, Extracellular Water; BCM, Body Cell Mass; BCMI, Body Cell Mass Index; FM, Fat Mass; FMI, Fat Mass Index; FFM, Fat-Free Mass; FFMI, Fat-Free Mass Index; SMI, Skeletal Muscle Index; ASMM, Appendicular Skeletal Muscle Mass; ASMMI, Appendicular Skeletal Muscle Mass Index.

Bold values refers to significant values.

For phase angle, the model-estimated increase was 0.244° between visits I and II (95% CI 0.063-0.425) and 0.280° between visits I and III (95% CI 0.069 - 0.491). BCM% increased by 1.482 percentage points between visits I and II (95% CI 0.405- 2.557) and by 1.796 percentage points between visits I and III (95% CI 0.541 - 3.050). ECW% decreased by 1.414 percentage points between visits I and II (95% CI -2.424 to -0.405) and by 1.663 percentage points between visits I and III (95% CI - 2.840 to -0.486). All six comparisons remained significant after FDR correction (FDR-adjusted p=0.016). No significant differences were observed between visits II and III for these parameters (**Figure 3**).

**Figure 3:**
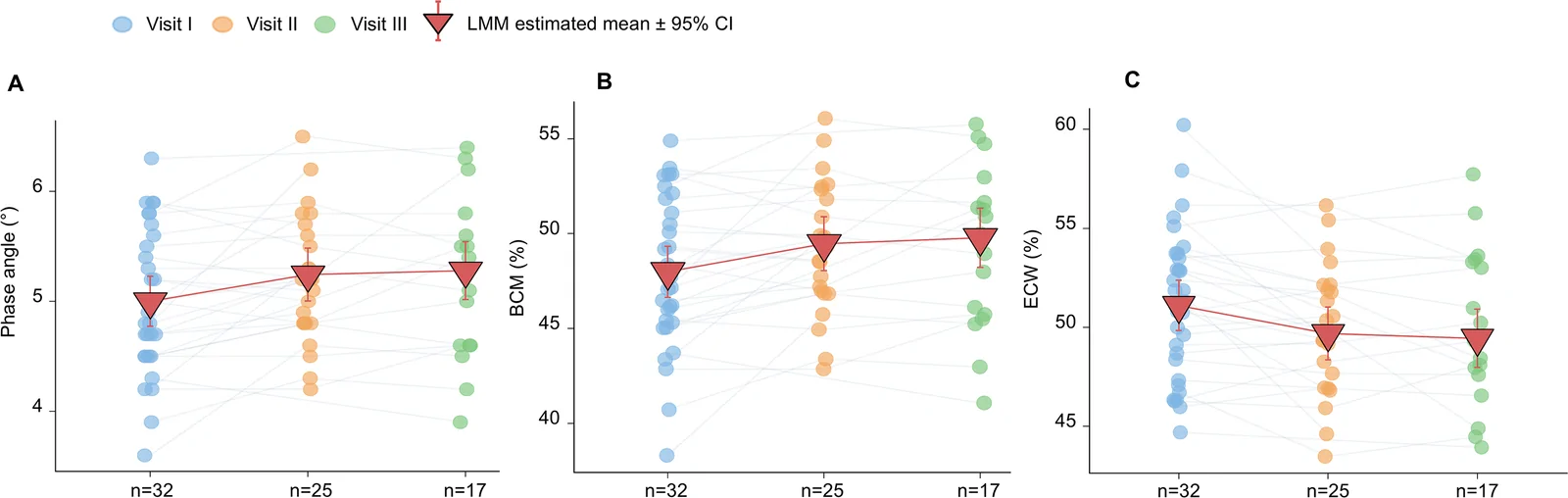
Longitudinal changes in selected BIA-derived parameters during nutritional follow-up. Individual observed values are shown as jittered points at each visit, while lines connect repeated measurements from the same patient across available visits. Red inverted triangles represent model-estimated marginal means at each visit, with vertical bars indicating 95% confidence intervals; the solid red line connecting the inverted triangles illustrates the trajectory of the model-estimated means across visits. Panels show phase angle **(A)**, BCM% **(B)**, and ECW% **(C)**. Statistical inference was based on separate linear mixed-effects models including visit as a categorical fixed effect and patient as a random intercept, using all available longitudinal measurements. Overall visit effects were adjusted for multiple testing using the Benjamini-Hochberg false discovery rate procedure.

In the exploratory analysis of raw impedance components, Xc/H showed a significant longitudinal change after FDR correction (FDR-adjusted p=0.037), whereas no significant overall changes were observed in R/H or impedance vector length (Z/H). Xc/H increased by 1.54 Ω/m between visits I and II (95% CI 0.47–2.62; FDR-adjusted p=0.015), with no further significant change between visits II and III. Thus, the early increase in phase angle was accompanied predominantly by a change in the reactive component rather than by a consistent change in resistance or overall vector length.

Complete-case sensitivity analyses were performed in the 14 patients with BIA measurements available at all three visits. Friedman tests showed unadjusted differences across visits for phase angle (χ²=8.75, p=0.013), BCM% (χ²=6.22, p=0.045), and ECW% (χ²=6.15, p=0.046). However, none of the 15 complete-case analyses remained statistically significant after FDR correction (**Supplementary Table S1**).

Overall, the mixed-effects analyses showed early longitudinal changes in phase angle, BCM%, and ECW%, followed by relative stability between visits II and III, whereas conventional indices of muscle quantity showed no statistically significant longitudinal changes over the observed follow-up period.

In mixed-effects analyses, body weight, BMI, and pain VAS showed significant overall visit effects after FDR correction (FDR-adjusted p=0.048 for each), whereas total daily PERT exposure did not (FDR-adjusted p=0.150). Compared with visit I, the estimated visit III changes were +1.69 kg for body weight, +0.61 kg/m² for BMI, and +12.55 points for pain VAS; pain VAS was also 9.94 points higher at visit II. However, none of the individual pairwise contrasts remained statistically significant after FDR correction across the nine comparisons (all FDR-adjusted p≥0.054,), (**Supplementary Table S2**).

## Discussion

This study provides a real-world snapshot of body composition in patients with CP referred for dedicated nutritional assessment due to clinical concern regarding nutritional status. Conventional measures were often relatively reassuring: mean BMI at the first nutritional consultation was 22.3 ± 3.8 kg/m² and 24 of 31 patients (77.4%) were classified as low nutritional risk by MUST. However, BIA provided a more nuanced picture. Low FFMI was present in 18.8% of patients, whereas low ASMMI was identified in 40.6%, indicating that reduced appendicular muscle quantity was not uncommon even though the cohort did not appear overtly malnourished according to BMI or MUST. This discrepancy supports the complementary role of body-composition assessment when clinical concern persists despite conventional screening (6).

The main longitudinal observation was an early change in PhA, BCM%, and ECW% during nutritional follow-up. PhA and BCM% increased and ECW% decreased by the second nutritional assessment, approximately three months after baseline, and the magnitude of these differences was maintained at the third visit. Body weight and BMI also showed modest overall visit effects in mixed-effects analyses, although no individual pairwise contrast remained statistically significant after FDR correction. Conventional indices of muscle quantity did not show statistically significant longitudinal changes. This pattern is compatible with the possibility that anthropometric and cellular/fluid-related BIA parameters may change over a shorter time frame than measurable changes in muscle quantity.

This observation is directionally consistent with nutritional-intervention data outside CP. In the real-world study by Cornejo-Pareja et al., malnourished or nutritionally at-risk adults receiving dietary counselling and ONS for up to six months showed improvements in nutritional status, body weight, and fat-free mass; in the subgroup with serial BIA, patients receiving an HMB-enriched high-protein supplement also showed increases in PhA and BCM (17). The population, supplement composition, and study design differed from the present cohort, so these findings cannot be directly extrapolated to CP. Nevertheless, they support further investigation of PhA and related qualitative BIA measures as potentially responsive markers during nutritional care.

Because phase angle may be influenced by hydration status, we additionally examined the raw impedance components. The early increase in phase angle was accompanied predominantly by an increase in Xc/H, whereas R/H and overall vector length did not show significant longitudinal changes. This pattern suggests that the observed phase-angle increase was unlikely to be explained solely by a generalized shift in body-water content and was directionally consistent with the concomitant increase in BCM% and reduction in ECW% (29).

Importantly, the longitudinal analyses did not identify an obvious clinical explanation for the observed BIA pattern. Total daily PERT exposure did not show a significant overall change during follow-up. Pain VAS showed an overall visit effect, but no individual pairwise contrast remained statistically significant after FDR correction, and there was no evidence of improvement in pain severity over time. Patients with previous alcohol exposure had already discontinued active alcohol abuse before entering nutritional follow-up, and no changes in analgesic treatment by the gastroenterology team were recorded during the nutritional follow-up interval. Although no significant overall change in PERT exposure or improvement in pain was observed, the contribution of concomitant clinical factors cannot be excluded.

PhA is of particular interest because it has repeatedly shown prognostic associations across chronic diseases and gastrointestinal and pancreatic settings, with lower values associated with adverse outcomes (30–32). Contemporary reference data also demonstrate substantial age- and sex-dependence of PhA (18), supporting our decision to analyze it as a continuous variable rather than apply a disease-external dichotomous threshold. BCM reflects the metabolically active cellular compartment, while ECW-related measures provide complementary information on fluid distribution and tissue quality. Taken together, these findings suggest that serial BIA may provide complementary information during nutritional follow-up in CP, particularly for short-term changes in cellular and fluid-related parameters that may not be captured by BMI or muscle-quantity indices alone. However, the present study should be interpreted as hypothesis-generating rather than as evidence of treatment efficacy.

Several limitations for this study should be acknowledged. First, BIA was not systematically performed in the entire BIOPANCREAS cohort but was requested according to routine clinical practice in patients for whom dedicated nutritional assessment was considered appropriate. This resulted in a selected study population and limits the generalizability of the findings. Second, the sample size was small and follow-up was incomplete; although mixed-effects models allowed all available longitudinal measurements to be included, missing follow-up data may still have introduced bias. Third, this was a single-centre observational study without a control group, and nutritional management was individualized rather than protocolized. Adherence was reassessed through dietary recall and interview but was not quantified with a standardized instrument. Therefore, the observed changes cannot be causally attributed to nutritional treatment, and regression to the mean, hydration variability, changes in disease activity, or other time-varying factors cannot be excluded.

Finally, follow-up was relatively short, with a median interval of approximately three months between assessments, which may have limited the detection of changes in conventional indices of muscle quantity.

Prospective controlled studies are required to establish whether serial BIA can reliably capture treatment-related changes and whether these changes are associated with clinically relevant outcomes.

## Data Availability

The datasets analyzed during the current study are available from the corresponding author upon reasonable request, in accordance with applicable institutional and ethical requirements. Requests to access the datasets should be directed to capurso.gabriele@hsr.it.
