# Supplementary material for: Serial bioelectrical impedance changes during nutritional follow-up in chronic pancreatitis
Source: Front Gastroenterol (Lausanne). 2026 Oct 20;5:1933330. doi: 10.3389/fgstr.2026.1933330 (PMC13727375; doi:10.3389/fgstr.2026.1933330)
Supplement: Supplementary file 1 [file Table1.docx]

**Supplementary Table S1. Complete-case sensitivity analysis of longitudinal BIA-derived parameters**

| **Outcome** | **Visit I Median (IQR)** | **Visit II Median (IQR)** | **Visit III Median (IQR)** | **Friedman χ²** | **Raw p** | **FDR-adjusted p** |
| --- | --- | --- | --- | --- | --- | --- |
| *Resistance (Ω)* | 551.25 (490.58–559.90) | 548.90 (482.88–595.30) | 516.35 (460.90–579.38) | 2.22 | 0.330 | 0.495 |
| *Reactance (Ω)* | 45.25 (39.95–50.95) | 48.00 (42.10–55.08) | 49.95 (40.30–52.55) | 2.37 | 0.306 | 0.495 |
| *Phase angle (°)* | 4.70 (4.50–5.57) | 5.00 (4.65–5.57) | 5.20 (4.60–5.57) | 8.75 | **0.013** | 0.189 |
| *TBW (%)* | 60.57 (55.50–63.36) | 58.02 (57.66–63.07) | 59.73 (55.82–63.16) | 0.69 | 0.708 | 0.817 |
| *ECW (%)* | 52.62 (47.59–53.98) | 50.81 (47.83–53.01) | 49.79 (47.73–53.22) | 6.15 | **0.046** | 0.231 |
| *BCM (%)* | 46.35 (45.12–51.65) | 48.26 (46.02–51.58) | 49.47 (45.84–51.56) | 6.22 | **0.045** | 0.231 |
| *BCMI (kg/m²)* | 8.18 (6.92–9.57) | 8.52 (7.42–9.68) | 8.59 (8.13–10.13) | 4.23 | 0.121 | 0.363 |
| *FM (%)* | 19.58 (15.65–24.26) | 21.03 (14.16–21.87) | 18.78 (14.33–24.05) | 0.76 | 0.683 | 0.817 |
| *FMI (kg/m²)* | 4.17 (3.13–5.79) | 4.37 (2.98–5.53) | 4.60 (3.21–5.35) | 0.33 | 0.849 | 0.910 |
| *FFM (%)* | 82.06 (75.74–85.22) | 78.97 (78.13–85.84) | 81.22 (75.95–85.67) | 0.11 | 0.947 | 0.947 |
| *FFMI (kg/m²)* | 17.37 (16.20–19.40) | 17.05 (16.29–19.70) | 18.38 (16.85–19.40) | 4.25 | 0.119 | 0.363 |
| *SM (kg)* | 23.20 (19.35–26.58) | 23.45 (19.33–26.55) | 23.30 (20.48–28.13) | 2.22 | 0.330 | 0.495 |
| *SMI (kg/m²)* | 8.46 (7.82–9.23) | 8.25 (7.63–9.48) | 8.50 (7.98–10.28) | 1.71 | 0.424 | 0.579 |
| *ASMM (kg)* | 16.90 (15.63–19.30) | 16.75 (15.85–19.70) | 17.35 (16.20–20.20) | 3.04 | 0.219 | 0.469 |
| *ASMMI (kg/m²)* | 6.36 (5.67–6.98) | 6.17 (5.72–7.07) | 6.44 (6.19–7.41) | 3.04 | 0.219 | 0.469 |

Values are median (IQR) for the 14 patients with BIA measurements available at all three visits. Overall differences across visits I, II, and III were assessed using Friedman tests. Benjamini-Hochberg FDR correction was applied across the 15 sensitivity analyses. No outcome remained statistically significant after FDR correction.

*Abbreviations: ASMM, appendicular skeletal muscle mass; ASMMI, appendicular skeletal muscle mass index; BCM, body cell mass; BCMI, body cell mass index; ECW, extracellular water; FDR, false discovery rate; FFM, fat-free mass; FFMI, fat-free mass index; FM, fat mass; FMI, fat mass index; SM, skeletal muscle mass; SMI, skeletal muscle mass index; TBW, total body water.*
