# Supplementary material for: Serial bioelectrical impedance changes during nutritional follow-up in chronic pancreatitis
Source: Front Gastroenterol (Lausanne). 2026 Oct 20;5:1933330. doi: 10.3389/fgstr.2026.1933330 (PMC13727375; doi:10.3389/fgstr.2026.1933330)
Supplement: Supplementary file 2 [file Table2.docx]

**Supplementary Table S2. Longitudinal mixed-effects analyses of body weight, BMI, pain severity, and PERT exposure**

**A. Model-estimated marginal means and overall visit effects**

| **Outcome** | **Visit I**  ***Estimated mean (95% CI)*** | **Visit II**  ***Estimated mean (95% CI)*** | **Visit III**  ***Estimated mean (95% CI)*** | **Overall raw p** | **Overall FDR-adjusted p** |
| --- | --- | --- | --- | --- | --- |
| *Body weight (kg)* | 62.70 (58.77 to 66.63) (n=32) | 63.83 (59.87 to 67.80) (n=25) | 64.39 (60.36 to 68.42) (n=17) | **0.035** | **0.048** |
| *BMI (kg/m²)* | 22.31 (21.07 to 23.55) (n=32) | 22.70 (21.45 to 23.96) (n=25) | 22.92 (21.64 to 24.21) (n=17) | **0.036** | **0.048** |
| *Pain VAS (0–100)* | 68.44 (59.45 to 77.42) (n=32) | 78.37 (68.35 to 88.40) (n=22) | 80.98 (69.44 to 92.52) (n=14) | **0.016** | **0.048** |
| Total daily PERT exposure (U/day) | 100.469 (80.244–120.694)  (n=32) | 101.398 (81.166–121.630)  (n=25) | 101.627 (81.383–121.871)  (n=17) | 0.150 | 0.150 |

**B. Model-based pairwise contrasts for outcomes with a significant overall FDR-adjusted visit effects**

| **Outcome** | **Contrast** | **Estimated difference** | **95% CI** | **Raw p** | **FDR-adjusted p** |
| --- | --- | --- | --- | --- | --- |
| *Body weight* | Visit II − I | +1.13 kg | −0.07 to +2.33 | 0.064 | 0.113 |
|  | Visit III − I | +1.69 kg | +0.29 to +3.09 | **0.018** | 0.054 |
|  | Visit III − II | +0.56 kg | −0.91 to +2.03 | 0.458 | 0.515 |
| *BMI* | Visit II − I | +0.39 kg/m² | −0.04 to +0.82 | 0.075 | 0.113 |
|  | Visit III − I | +0.61 kg/m² | +0.11 to +1.12 | **0.017** | 0.054 |
|  | Visit III − II | +0.22 kg/m² | −0.31 to +0.75 | 0.408 | 0.515 |
| *Pain VAS* | Visit II − I | +9.94 | +1.31 to +18.56 | **0.024** | 0.054 |
|  | Visit III − I | +12.55 | +2.20 to +22.89 | **0.017** | 0.054 |
|  | Visit III − II | +2.61 | −8.64 to +13.86 | 0.649 | 0.649 |

All four longitudinal outcomes were analyzed using separate linear mixed-effects models fitted using restricted maximum likelihood (REML), with visit as a categorical fixed effect and patient as a random intercept. Analyses were restricted to visits with concomitant BIA. For PERT exposure, patients documented as not receiving PERT were assigned 0 U/day. Benjamini-Hochberg FDR correction was applied across the four overall visit tests. Pairwise model-based contrasts were performed only for outcomes with a significant overall FDR-adjusted visit effect, with FDR correction applied across the nine resulting pairwise contrasts. PERT use at BIA visits was 29/32 (90.6%), 23/25 (92.0%), and 14/17 (82.4%) at visits I, II, and III, respectively.

*BMI Body Mass Index; VAS Visual Analogue Scale; PERT Pancreatic Enzyme Replacement Therapy*
